# Fabrication and Validation of Sub-Cellular Carbon Fiber Electrodes

**DOI:** 10.1109/TNSRE.2024.3360866

**Published:** 2024-02-13

**Authors:** Julianna Richie, Joseph G. Letner, Autumn McLane-Svoboda, Yu Huan, Dorsa Haji Ghaffari, Elena della Valle, Paras R. Patel, Hillel J. Chiel, Galit Pelled, James D. Weiland, Cynthia A. Chestek

**Affiliations:** Department of Biomedical Engineering, University of Michigan, Ann Arbor, MI 48105 USA; Department of Biomedical Engineering, University of Michigan, Ann Arbor, MI 48105 USA; Department of Biomedical Engineering, Michigan State University, East Lansing, MI 48824 USA; Department of Biology, Case Western Reserve University, Cleveland, OH 44106 USA; Department of Biomedical Engineering, University of Michigan, Ann Arbor, MI 48105 USA; Biointerface Institute, Department of Biomedical Engineering, University of Michigan, Ann Arbor, MI 48105 USA; Department of Biomedical Engineering, University of Michigan, Ann Arbor, MI 48105 USA; Department of Neuroscience, Department of Biomedical Engineering, Department of Biology, Case Western Reserve University, Cleveland, OH 44106 USA; Department of Mechanical Engineering, Neuroscience Program, Department of Radiology, Michigan State University, East Lansing, MI 48824 USA; Department of Biomedical Engineering, Department of Ophthalmology and Visual Sciences, University of Michigan, Ann Arbor, MI 48109 USA; Department of Biomedical Engineering, Department of Electrical Engineering and Computer Science, Department of Robotics, University of Michigan, Ann Arbor, MI 48105 USA

**Keywords:** Carbon fiber, small pointed fiber electrode (SPFe), foreign body response (FBR), Platinum Iridium (PtIr), scanning electron microscope (SEM), cyclic voltammetry (CV), electrical impedance spectroscopy (EIS), high-density carbon fiber (HDCF), retinal ganglia cell (RGC), axial nerve cord (ANC), cathodic charge storage capacity (CSCc)

## Abstract

Multielectrode arrays for interfacing with neurons are of great interest for a wide range of medical applications. However, current electrodes cause damage over time. Ultra small carbon fibers help to address issues but controlling the electrode site geometry is difficult. Here we propose a methodology to create small, pointed fiber electrodes (SPFe). We compare the SPFe to previously made blowtorched fibers in characterization. The SPFe result in small site sizes (105.4 ± 20.8 *μ*m^2^) with consistently sharp points (20.8 ± 7.64°). Additionally, these electrodes were able to record and/or stimulate neurons multiple animal models including rat cortex, mouse retina, *Aplysia* ganglia and octopus axial cord. In rat cortex, these electrodes recorded significantly higher peak amplitudes than the traditional blowtorched fibers. These SPFe may be applicable to a wide range of applications requiring a highly specific interface with individual neurons.

## I. INTRODUCTION

AWIDE range of medical and clinical therapies rely on stimulating or recording neural tissue using implantable electrodes. For example, deep brain stimulation (DBS) has been used in over 160,000 people to treat Parkinson’s related tremor and gait irregularity [1]. Recording with implantable electrodes is less common in clinical applications. However, there are emerging applications, including those that use recorded neural signals as biomarkers to modulate stimulation. For example, the RNS system (Neuropace, Mountain View, CA) detects pre-ictal activity to trigger neural stimulation to treat epilepsy [2].

The electrodes used to record and stimulate in those clinical applications interact with populations of neurons instead of individual neurons due to their large surface area. These particular therapies can provide benefit to patients despite their lack of neuron specificity. However, large electrodes cannot be used for therapies where targeting small groups of cells is a requirement. For example, in retina stimulation to provide vision for the blind, stimulating retinal ganglion cells (RGC) with a small electrode is required to activate only a small number of cells for the highest visual acuity [3]. Similarly, as a recording application, brain machine interfaces involve recording from specific neurons and interpreting their activity to provide commands for assistive technology [4]. Neuron level resolution enables differentiation between the activity representing different fingers, intended velocity, and amount of force translated from brain to arm [5], [6]. To fully isolate a single unit, an electrode needs to be similarly sized (100 *μ*m^2^) and close to the neuron [7]. Low impedance is likely required to have sufficiently low noise but this is challenging to achieve on small electrodes as surface area and impedance are inversely related.

Most electrode arrays designed to interface with individual neurons have been designed with small electrode sites on stiff substrates. Due to its widespread use in electronics, silicon devices are easiest to manufacture and many electrodes can be densely packed on a single wafer [8], [9]. Notably, the Utah Electrode Array (UEA) has been used for many years in humans and nonhuman primates with multi-year recordings [10], [11], [12], [13]. Planar shank-based designs (e.g., Neuropixel [14], Cambridge [15], NeuroNexus [16]) allow for simultaneous recording from multiple cortical layers, whereas the UEA places all electrode sites at one depth. But high-resolution planar shank-based electrode arrays are not routinely used as chronic electrodes in humans or NHP due to the planar geometry. The most recent silicon electrodes have substantially increased the number of channels from 100 on to close to 1000 [14]. However, silicon substrate arrays may fundamentally cause tissue damage [17], [18], continuous inflammation around the device sites, and counterproductively, a decrease in the neuron density around the recording site [18], [19], [20], [21], [22]. These issues motivate the investigation of other materials to preserve the tissue while maintaining the ability to record selectively from neurons.

Ideally, electrodes should cause very little foreign body response (FBR), and several strategies have been attempted to achieve this. For example using materials with lower Young’s modulus (softer) may lead to a lower FBR [22], [23], [24], [25]. Reducing electrode substrate size to cellular sizes also leads to lower FBR, even when using stiffer materials [26], [27]. Additionally, the geometry can reduce the force needed to implant into brain that can cause initial damage. However, it is difficult to make robust probes from silicon alone at these small sizes. Therefore, cellular scale probes have been fabricated primarily using other materials. The Net10/50 probes [28], [29] and amorphous silicon carbide “ultramicroelectrodes” [30], both utilize thin, flexible shanks that cause little insertion damage while still recording high quality signals. Specifically, the Net10 probes use SU-8 and lithography techniques to create a 10 *μ*m × 1.5 *μ*m cross-section. However, soft devices often require specialized insertion techniques, such as an insertion shuttle or a stiffening agent [25], [31], [32]. While many soft probes boast small cross-sectional areas, in practice wider devices are used more often than the smallest form factors due to durability.

Another limitation of putting lots of contacts along a shank is their confinement to a column of neurons. Many applications require recording capabilities across a larger brain area.

For these applications, microwire arrays are a popular design as they are both commercially available and can also be fabricated by individual labs. These arrays have diameters down to 30–50 *μ*m, and even down to 5 *μ*m in research settings [33]. However, metal microwires tend to deform upon insertion [34], which causes unwanted tissue damage. More importantly, the deformation removes confidence in the placement of the electrodes until after the tissue can be imaged. As these microwires deform easily at these scales [35], [36], stiffeners [25] can be used to achieve more reliable placement. For example, the CHIME array [37] utilizes glass insulation to create a multi-electrode array that can hold its shape. While glass is sufficiently stiff,it is brittle and breaks easily under stress preventing it from being a reliable chronic implant [38]. Additionally, microwires are known to corrode *in vivo* causing cracks to form in the insulation and at the electrode degrading the array’s performance [39].

Carbon arrays have become a viable option [40], [41], [42], [43], [44], [45] due to carbon’s inherent strength and conductivity. Carbon arrays have been implemented in a number of form factors. Carbon yarn [46], [47], [48] has been shown to record in nerve, and can be assembled in the lab with 10–30 *μ*m diameters [46], [48]. To enable deep penetration into brain 64 fibers (7 *μ*m diameter) were combined into a 200 *μ*m diameter electrode [44]. However, these approaches do not allow for distributed array recordings across a large area. Our group has worked towards multi-channel arrays where carbon fibers are distributed over a larger substrate, where each fiber acts as an independent recording or stimulation channel [49], [50]. In previous work, we have demonstrated a carbon fiber array insertion into brain with individuated fibers for single unit recordings at up to 80 um pitch. We have also investigated modifying the fibers for insertion into tougher tissues like nerve and deeper unassisted insertion into cortex. These applications require a sharpened fiber [45], [51], but the sharpening process left the fibers with relatively large recording sites that cannot readily isolate single units.

In this paper, we describe a fabrication process for “small, pointed fiber electrodes” (SPFe) that provide both small electrode surface area for single unit recording and sharpened tips for better penetration into tissue. We use chemical etching of carbon fibers to give the SPFe a repeatable ~100 *μ*m^2^ electrode area without loss of electrode functionality. The approach is based on that used for fabricating carbon fiber microscopy tips used in imaging [52], [53], [54]. Here, we evaluate the electrochemical and physical properties of SPFe and deposit conductive coatings to reduce impedance and enable microstimulation despite this small size. Finally, we demonstrate SPFe in multiple biological preparations to demonstrate feasibility. We show here that chemical etching is an appropriate and robust method for optimizing the carbon fiber electrode tip to sub-cellular size capable of recording and stimulating across multiple tissue types and animal models.

## II. METHODS

### A. Probe Fabrication

**Fig. 1.**
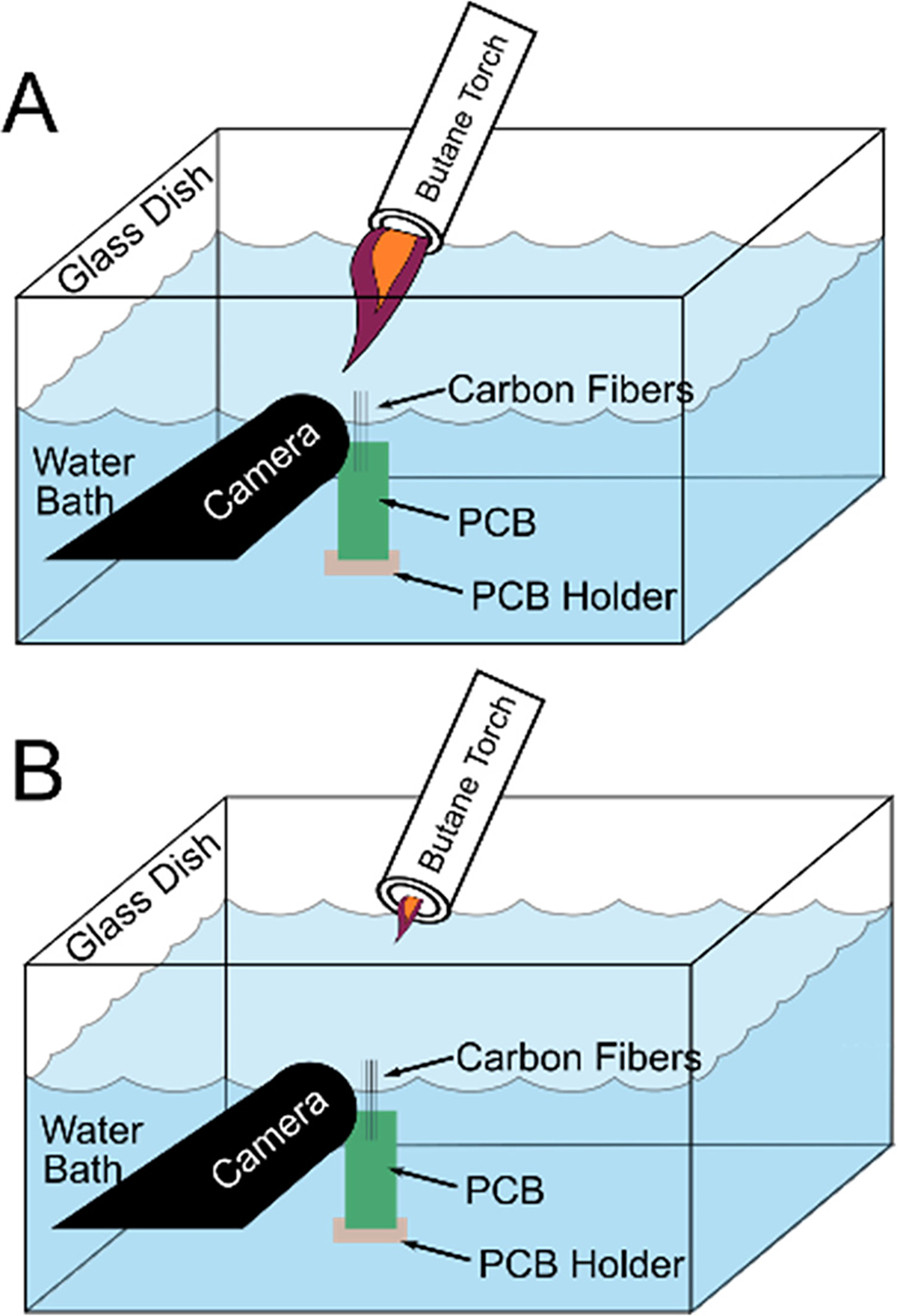
Large-blowtorch (A) vs small-blowtorch (B methods). The water bath is set up the same way, but the small-blowtorch flame is much smaller than the flame in A. The smaller flame allows for a smaller tip to be exposed (< 100 *μ*m).

Carbon fiber arrays were fabricated following similar methodologies as previously reported [49], [50]. Briefly, a printed circuit board with exposed gold traces was coated with silver conductive epoxy (H20E, Epoxy Technology, Billerica, MA) and carbon fibers were inserted into the silver epoxy. The epoxy was cured then insulated using a non-conductive epoxy ((NOA61;NorlandProducts,Inc., Cranbury,NJ). Arrays were coated with Parylene C (Parylene C Deposition System 2035, Specialty Coatings Systems, Indianapolis, IN) to a thickness of ~ 800 nm. The carbon fibers were then blowtorched [45] (Figure 1) to remove Parylene C from the tips and re-expose a small portion of the carbon fiber. One set of fibers (n = 69) was exposed with a larger flame (Figure 1 A) exposing ~140 *μ*m of carbon. This group was further processed using chemical etching (SPFe). Another group (n = 47) used a small blowtorch flame (Figure 1 B) to expose < 100 *μ*m of the carbon fiber from Parylene C. For clarity, the groups will be referred to as large-blowtorch (~140 *μ*m exposure, using historical data [45] n = 574), SPFe (chemical etch), and small-blowtorch (< 100 *μ*m exposure). HDCF arrays were prepared for implant following previous literature [55] for ease of surgeon use. For HDCFs in this study, every other fiber was SPFe or large-blowtorch for easy comparison of the two electrode preparations.

To lower impedance of the electrodes a coating of PEDOT:pTS or Platinum Iridium (PtIr) was applied to the electrode following the methods in previous literature [50], [56], [57]. Simply, recording electrodes were coated with a solution of 0.1M:0.01M PEDOT:pTS by applying 600 pA current to each electrode for 10 minutes. PtIr was applied using a solution of 0.2 g/L Na3IrCl6H2O and 0.186 g/L Na2PtCl6H2O in 0.1 M nitric acid and applying a CV sweep from −0.1 to 0.1 V at 200 mV/s for 250–300 cycles depending on the size of the exposed carbon fiber.

### B. Chemical Etching

**Fig. 2.**
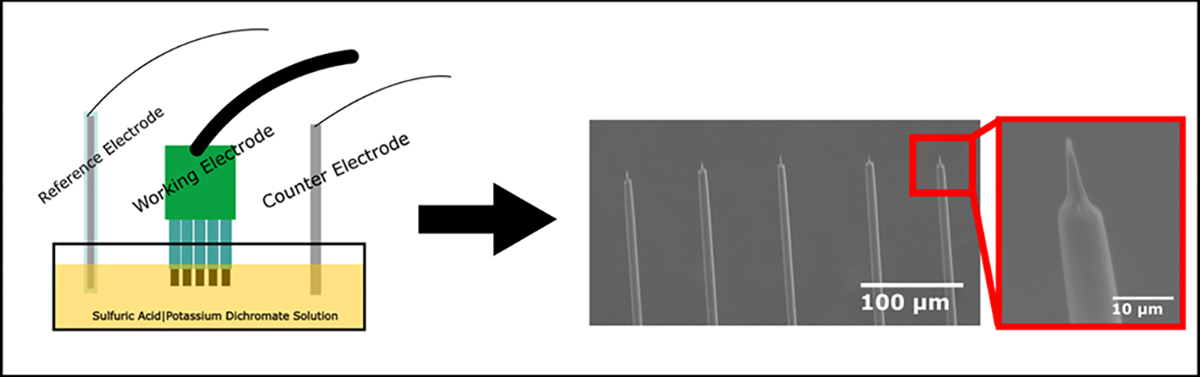
Schematic showing the etching set up (left) and resulting sharpened tips (right). Set up for etching is simple with a reference and counter electrode submerged in solution with the exposed tips of the carbon fiber array. A voltage pulse is applied for several seconds and then the fiber tips are imaged to check geometry. The tips are sharpened and sub-cellular in size as seen in the far right image.

A solution of 0.5 mM K_2_Cr_2_O_7_|5 M H_2_SO_4_ was prepared to etch the tips of the carbon fibers [53], [54]. Following previous literature [54], the SPFe were lowered into the solution along with a reference and counter electrode (described in Electrical Characterization) (Figure 2). A constant 3.5 V_rms_ was applied in one-second increments for no more than a cumulative 4 seconds. The probes were triple rinsed in DI water and allowed to rest for a minute after each 1 second etch to help disperse the built-up surface charge on the electrode before measuring 1 kHz impedance in PBS solution. Scanning electron microscopy (SEM) confirmed a small, pointed tip was reliably attained once an impedance increase of at least 1 MΩ (at 1 kHz) after etching was measured. This relative impedance increase of 1 MΩ from the un-etched impedance occurred between 2–4 seconds of etching for all fibers in the study.

### C. Physical Characterization

Tip length and sharpness were quantified using scanning electron microscopy (SEM) imaging (Tescan Rise, Tescan Orsay Holding, Brno—Kohoutovice, Czech Republic). SEM imaging was performed under low vacuum mode (LVSTD, low vacuum secondary electron Tescan detector) to preserve electrode functionality. An excitation voltage between 5 and 20 kV was used. Tip length was defined as the distance between the tip of the exposed carbon to the lowest edge of the Parylene C transition. Sharpness was measured using the built in angle tool in the SEM software. The surface area of a cone was calculated using the measured height, h, and r=3.5μm:

(1) $$SA=\pi \cdot r*\sqrt{h^{2}+r^{2}}$$

### D. Insertion Testing

Insertion tests were performed following previous methodologies [50] in perfused rat brain. In short, a rodent brain perfused with 1x PBS was exposed and dura and pia were removed. A set of SFPe (0.5 mm – 5 mm, 0.5 mm increments) were lowered using a stereotaxic manipulator. Fibers were recorded as successful if they were able to penetrate into the brain and continue insertion along their full length. Unsuccessful insertion was determined by a continuous buckling of the fiber that never penetrated. Results were analyzed using a Wilson Binomial Confidence Interval Test to determine the 95% confidence intervals around each data point. Data was compared to historical data [50] using a Pearson’s Chi-Squared Test to determine significance.

### E. Electrical Characterization

All electrodes were subjected to cyclic voltammetry (CV) and electrochemical impedance spectroscopy (EIS) during different stages of the fabrication process. Impedances of the bare fibers were taken at several points during fabrication to ensure electrical connection and verify exposed area: after flame exposure, throughout the chemical etch process, and to ensure good coating of any added conductors. Following previous methods [50], a 1 kHz impedance scan was conducted in 1x PBS (BP3994, Fisher, Waltham, MA) with an Ag|AgCl reference electrode (RE-5B, BASi, West Lafayette, MA) and a 2 mm diameter, 3 cm long, hollow stainless-steel rod as the counter electrode. A PGSTAT12 Autolab potentiostat (EcoChemie, Utrecht, Netherlands) using NOVA software provided by the vendor was used to run the EIS and CV measurements. Results were analyzed using custom MATLAB scripts (MathWorks, Natick, MA) and reported as ‘mean ± standard deviation’.

Full-spectrum EIS was performed in a frequency range from 31 kHz to 10 Hz applying a 10 mV_rms_ sine wave following previous methods [50]. All EIS were performed in 1x PBS solution in the three-electrode configuration following the 1 kHz measurements above.. Cyclic voltammetry was taken only at the end of the tip preparation and before and after coating. This was done to monitor and characterize any redox reactions and to ensure a safe water window for each group of probes for use *ex vivo*. CV scans were obtained by sweeping three times between −0.6 and 0.8 V versus Ag|AgCl at a scan rate of 1 V/s. Charge storage capacitance (CSC_C_, [5]) was estimated from the CV data using the custom Matlab script.

### F. In-Vivo and In-Vitro Validation

All animal procedures were approved by the Institutional Animal Care and Use Committees at the University of Michigan or Michigan State University. Several animal models were used to investigate the modularity of the SPFe tips in different tissue types: rat motor cortex (softer tissue), mice RGCs (small cell), octopus axial nerve cord (ANC) (tough tissue), and A *plysia* ganglia (large cell).

#### 1) Rat Motor Cortex:

*In vivo* validation of SPFe tip recording capacity in rat cortex closely followed our previously reported terminal procedures performed to acutely measure electrophysiology [45], [56], [58]. We implanted high-density carbon fiber (HDCF) electrode arrays [59] with SPFe tips into adult male Long-Evans rats (n=2) weighing 330 & 370 g. Each rat was implanted with two electrode arrays, where the first was removed prior to implanting the second. In one rat, the electrode arrays had alternating large-blowtorch [45] and SPFe tips for direct comparisons of single unit amplitudes *in vivo*. Rats were briefly anaesthetized with isoflurane (5% v/v) to facilitate intraperitoneal injection of ketamine/xylazine (90/10 mg/kg) for anesthesia induction, which was maintained with periodic update injections of ketamine (30 mg/kg). Carprofen (5 mg/kg) was administered subcutaneously as an analgesic. Breath rate and temperature were monitored throughout the procedure. After an incision along the head’s midline, clearing the periosteum, and cleaning the skull, a stainless steel bone screw (cat. # 1ZY93, Grainger, Lake Forest, IL) was screwed through the skull, posterior to lambda, to touch the brain as an electrical reference. A craniotomy was drilled into the right hemisphere 1–3.5 or 1–2 mm mediolaterally (M/L) and 1–3.5 or 1–3 mm anteroposteriorly (A/P) relative to bregma targeting motor cortex. The first array was lowered via a stereotaxic arm to the dura surface to zero the probe coordinates on the dorsoventral axis followed by a durotomy and connecting a reference wire on the probe to the bone screw. Each probe was inserted to multiple depths spanning layers I – V of the motor cortex (0–1600 *μ*m, [60], [61]) and electrophysiology was recorded at each depth. for 3 minutes using a ZC16 headstage, RA16PA pre-amplifier, and RX7 Pentusa base station (Tucker-Davis Technologies, Alachua, FL) in a faraday cage with a sampling frequency of 24414.1 Hz. Rats were euthanized at the end of the procedure.

#### 2) Mouse Retina:

Intraretinal stimulation was performed using SPFe in *ex vivo* retina obtained from (C57BL/6) mice. The eyes were previously injected with rAAV2-CAG-GCaMP6f-WPRE-bGH to express calcium indicator GCaMP6f in retinal ganglion cells (RGC) [62], [63]. Three to four weeks after intraocular injection of the AAV vector, animals were euthanized with ketamine (100 mg kg^−1^) and xylazine (10 mg kg^−1^). Retinas were isolated, mounted on a transparent chamber, and superfused with bicarbonate-buffered Ame’s Medium to ensure cell health throughout the experiment. Carbon fiber electrodes were inserted from the photoreceptor side of the retina and calcium imaging was performed from the RGC side. The electrode tip was positioned at 20 *μ*m distance from the RGC layer vertically using a micromanipulator, using the baseline fluorescence of the RGCs to locate the RGC layer. Electrical stimulation was delivered by the PlexStim electrical stimulator (Plexon Inc., Dallas, TX) and the voltage transient across the electrodes was recorded by an oscilloscope connected to the voltage transient (VT) output of the PlexStim. Cathodic-first biphasic pulses with 100 *μ*s duration per phase were delivered at 120 Hz and various current amplitudes (5 – 15 *μ*A).

#### 3) Aplysia Ganglia:

The SPFe was tested in *Aplysia* neurons for its intracellular recording ability, following the method in Huan et al., 2021 [55]. Briefly, the buccal ganglia were isolated from the animal and were pinned to the Sylgard base of a dish. One buccal ganglion was carefully desheathed to expose individual neurons. To obtain an intracellular action potential, a SPFe fiber was inserted into a neuron until it penetrated the cell membrane. A glass microelectrode was inserted into the same neuron after the SPFe insertion to compare the recordings. To provide a direct comparison, both electrodes were connected to a DC-coupled intracellular amplifier (A-M Systems Model 1600, Everett, WA) and the signals were recorded in AxoGraph X (AxoGraph Scientific, Foster City, CA) at a sampling rate of 5000 Hz. The impedance of the glass microelectrode was 3.4 MΩ.

#### 4) Octopus Axial Nerve Cord (ANC):

Adult specimens of *Octopus bimaculoides* collected from the California coast were used to validate the insertion and recording capabilities of the electrodes [64]. The left front arm was amputated from the octopus body and placed in a dissection tray perfused with filtered saltwater from the housing tank for longevity. Dissection of the arm to isolate the ANC, a structure similar to that of the spinal cord, ensured that recordings would be only of neuronal activity. The SPFe was inserted directly into the ANC tissue at the base of the octopus arm for recording. To elicit activity, mechanical stimulation at the distal portion of the arm was performed. Recordings were taken through Spike2 (Cambridge Electronic Design Limited, Cambridge, England) software sampling at 30 kHz.

### G. Motor Cortex Spike Analysis

The recordings of the SPFe tips in rat cortex were analyzed using our previously described protocol [56], [65]. Briefly, electrophysiology was first common average referenced [66] in MATLAB 2022a (MathWorks, Natick, MA). Signals were then loaded into Plexon Offline Sorter (version 3.3.5) (Plexon Inc., Dallas, TX) and high-pass filtered (250 Hz cutoff, 4^th^ order Butterworth filter). A trained operator estimated the RMS baseline noise (V_rms_) for each channel by manually identifying five segments per channel that were 100 ms in length with low neural activity and low artifact noise. Thresholds were set at −3.5× V_rms_. To remove noise waveforms, cross-channel artifacts were invalidated, prospective clusters were manually selected in principal component analysis (PCA) space, waveforms assigned with K-Means clustering, and clusters containing clear noise clusters were invalidated. The remaining waveforms were clustered using Plexon Offline Sorter’s Standard Expectation-Maximization Scan function [56], after which oversorting and undersorting were corrected manually, and clusters were cleaned of remaining noise. Sorted waveforms were exported back to MATLAB for analysis using custom scripts.

### H. Retina Stimulation Data Analysis

Carbon fibers were inserted into the mouse retina as described above. A stimulation pulse train was delivered for 5 seconds. The recorded images of the calcium transient were then analyzed in MATLAB. Baseline fluorescence images were subtracted from images recorded during stimulation to obtain RGC spatial activity. This result was further normalized relative to baseline to account for the noise in the fluorescence signal.

### I. Aplysia Ganglia Spike Analysis

Recorded intracellular spike trains were loaded into MATLAB. The same action potentials recorded by the glass electrode and the SPFe were analyzed. To visualize the relative size of the spikes recorded by the two electrodes, the action potentials were superimposed on one other. To compare the sizes, the average amplitude of the spikes was calculated. Because the insertion of the electrode could disturb the cell membrane and trigger a train of action potentials, only action potentials after the train were used for the amplitude calculation. Only the last few action potentials within the train were used to determine the stabilized amplitude after insertion. Spike amplitudes are shown as ‘mean ± standard deviation.’

### J. Octopus ANC Spike Analysis

**Fig. 3.**
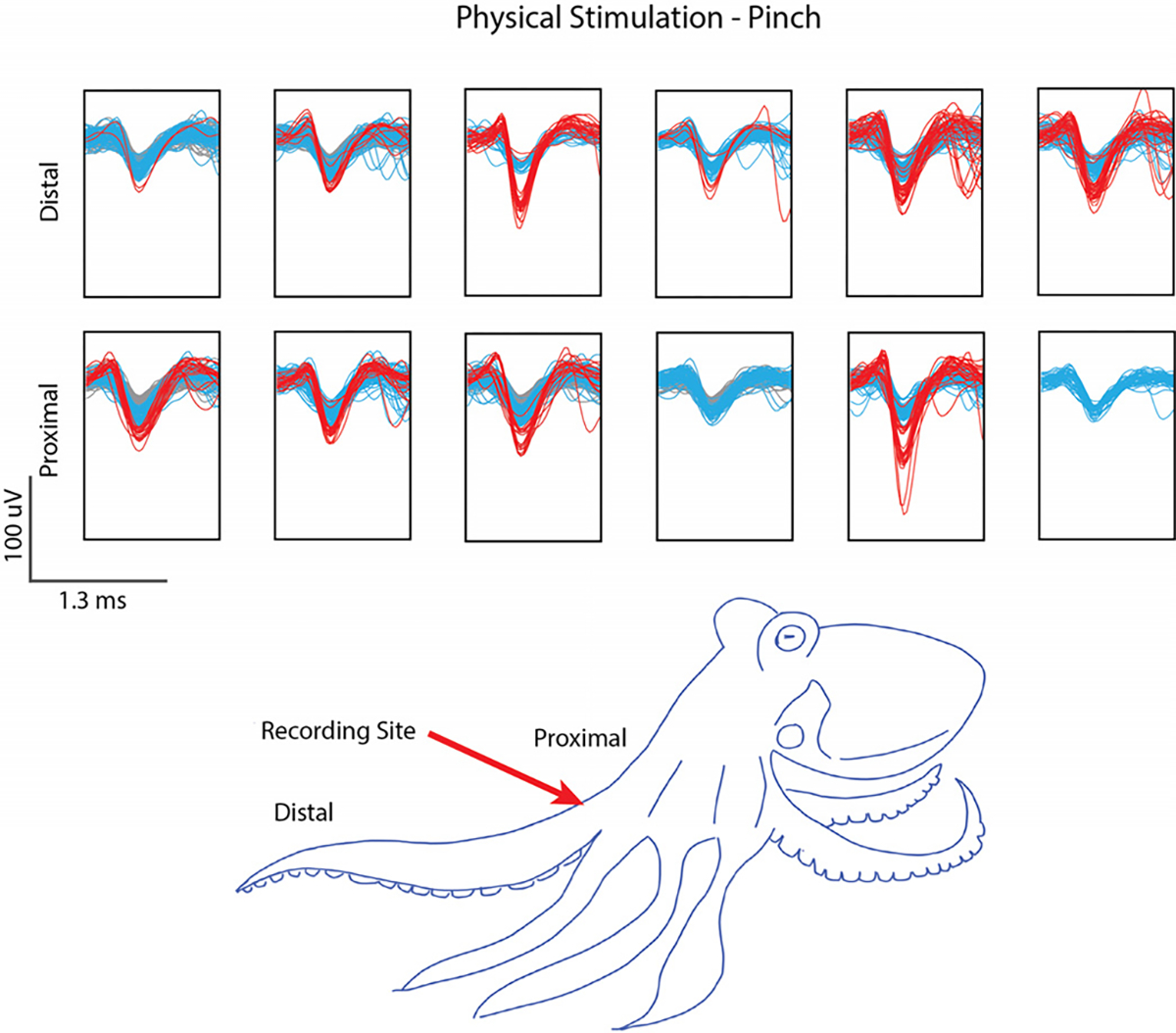
Spike panels from octopus arm. Arms were stimulated at the proximal and distal locations and the electrode array recorded at the proximal location. The gray lines represent waveforms with thresholds of < −20 *μ*V, blue −20 to −40 *μ*V, and red > 40 *μ*V. Red arrow indicates the recording site.

As Spike2 does not allow for the same spike sorting protocols used in Plexon, potential units were identified in MATLAB after waveform sorting in Spike2. A bandpass filter (0.1 to 3 kHz, 2^nd^ order Butterworth) was run on each channel and spikes were detected using a −4 × Std threshold. Spikes were automatically sorted into templates based on shape and clustered utilizing principal component analysis. Identified waveforms were exported to MATLAB. The spikes were color-coded and plotted to indicate 3 potential units using thresholds arbitrarily set at −40, −20, and 0 *μ*V (Figure 3).

## III. RESULTS

### A. Physical Characterization

**Fig. 4.**
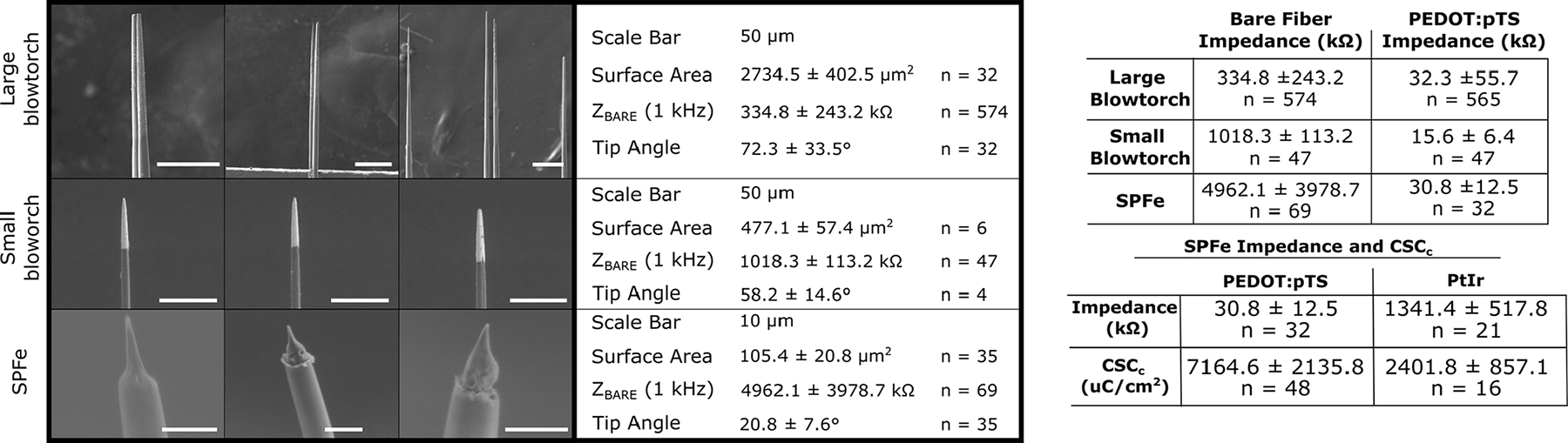
Example images from each type of sharpening method (left) with accompanying physical characteristics. Tables on the right show the 1 kHz impedance characterization across electrode tip geometry (top) as well as impedance and charge storage capacity across coating type (bottom).

To fabricate electrode tips that are sharp and small, we evaluated a chemical etching technique used in imaging literature [53], [54]. We compared SPFe to two blowtorch sharpened carbon fibers (large-blowtorch and small- blowtorch) [45], [65]. Figure 4 shows representative tips from these groups and characteristics reported as ‘mean ± SD.’ SPFe tips were generally sharp and consistent in size. To quantify the surface area exposed, SEM images were obtained (Figure 4). SPFe had an average surface area of 105.4 ± 20.8 *μ*m^2^ (n = 35). Compared to the large-blowtorch (2734.5 ± 402.5 *μ*m^2^, n=32 [56]) and the small-blowtorch (477.1 ± 57.4 *μ*m^2^, n=6) the SPFe are much closer to cellular sizes. Additionally, the angle of sharpening was 20.8 ± 7.64° (n = 30) for SPFe, 58.2 ± 14.59° (n = 4) for small-blowtorch, and 72.3 ± 33.5° (n = 32) [45] for large-blowtorch suggesting that chemical etching generates a sharper point.

To determine if blowtorching alone could achieve the small surface area of SPFe without chemical etching, a small-flame blowtorch was used to sharpen the carbon fiber tips (Figure 1 B). While the small-blowtorch was able to produce smaller tip geometries than large-blowtorch, the resulting tips were not sub-cellular in size; the smallest recorded height was 30 *μ*m. Moreover, the yield was low, often requiring an hour of effort per array. In contrast, chemical etch provided consistent sharpening after 1–4 seconds.

### B. Electrical Characterization

Given the smaller electrode site sizes, we wanted to determine whether the impedance remains at a level comparable to other cellular scale neural probes. First, 1 kHz impedance measurements were taken before both tip treatments to establish a baseline prior to any tip coatings that may increase the effective surface area. Predictably, large-blowtorched fibers had the lowest 1 kHz impedance (~300 k*n*Ω) followed by small-blowtorch fibers (~1MΩ), and SPFe fibers had the largest (> 4 MΩ) (see Figure 4 for specific values). We then applied a conductive coating to all geometries to lower the impedance and improve recording and stimulation performance.

Specifically for recording, we applied PEDOT:pTS, which has a complex topography that impedance does not necessarily scale directly with area [67]. Applying this to the electrodes lowered the impedances to similar values in the 10s of kiloOhms despite their differences in surface area (Figure 4). These values are in an appropriate range for effective single-unit recordings.

**Fig. 5.**
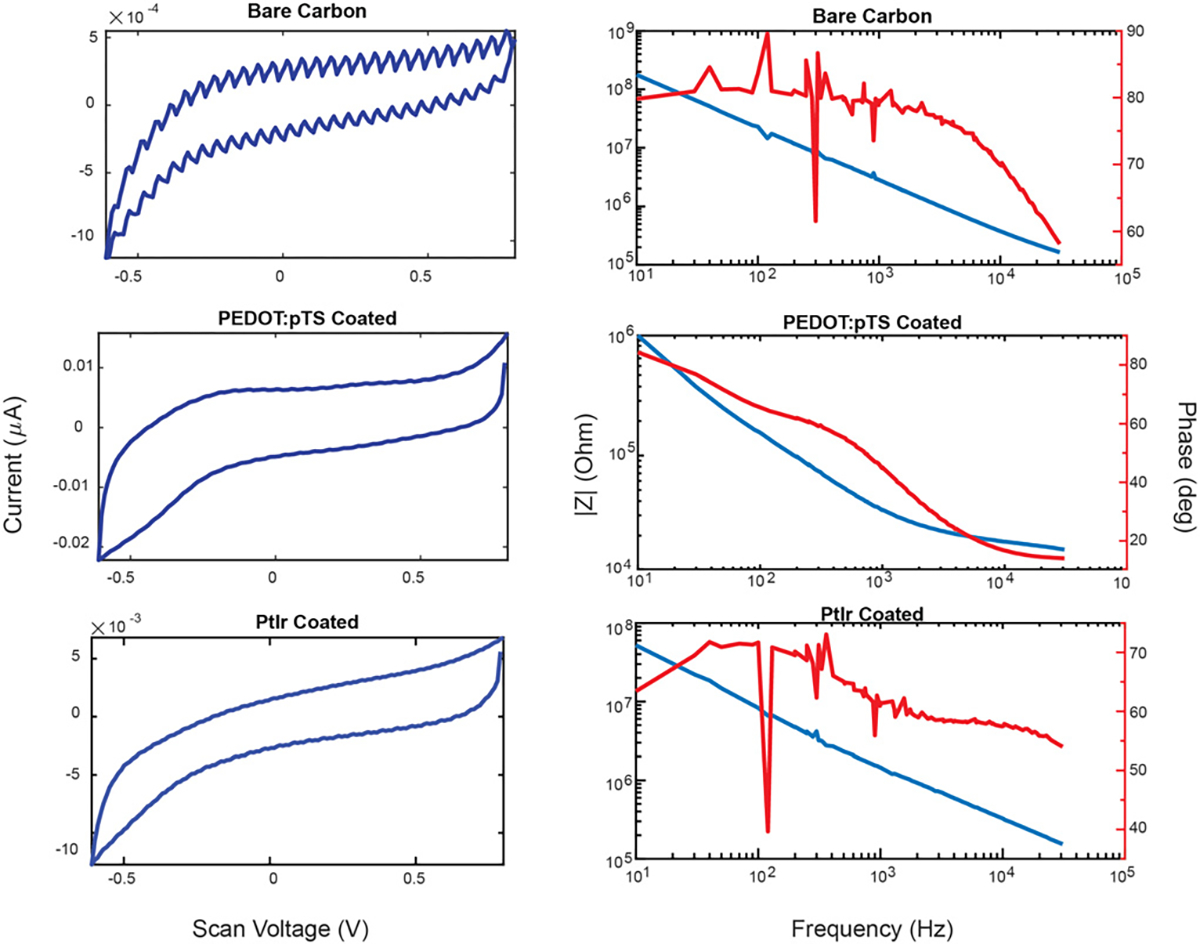
Cyclic voltammetry (left) and bode magnitude and phase (right) for a non-coated„ PEDOT:pTS coated, and PtIr coated SPFe. Notice the change in scale along the Y-axis for both sets of plots.

To determine the viability of the SPFe as a stimulation electrode, a different set of electrodes were coated with PtIr, which is more stable for stimulation than PEDOT:pTS [55]. Cyclic voltammetry, EIS, and voltage transient of a PtIr functionalized electrode were analyzed. SPFe cyclic voltammograms (Figure 5) showed very small deflections in their redox peak. The calculated cathodic charge-storage capacity (CSC_C_) for these SPFe when coated in either PEDOT:pTS or PtIr are reported in Figure 4. Briefly, CSC_C_ resulted in ~7000 *μ*C/cm^2^ after PEDOT:pTS coating and ~2000 *μ*C/cm^2^ after PtIr coating. Impedance for these PtIr functionalized SPFe at 1 kHz was 1341.4 ± 517.8 kΩ (n=21), which does scale appropriately to its associated surface area when compared to large-blowtorch electrodes (344 ± 16.9 kΩ, n = 70) as noted in previous literature [68]. While there is a large variability in the capacitance and impedance for these electrodes before coating, the plating step tends to act as a cleaning step [69] which helps to lower the variability post-coating in both PEDOT:pTS and PtIr cases. Also, the SPFe shape has variablility, which is reflected in the impedance data.

### C. Insertion Trials

**Fig. 6.**
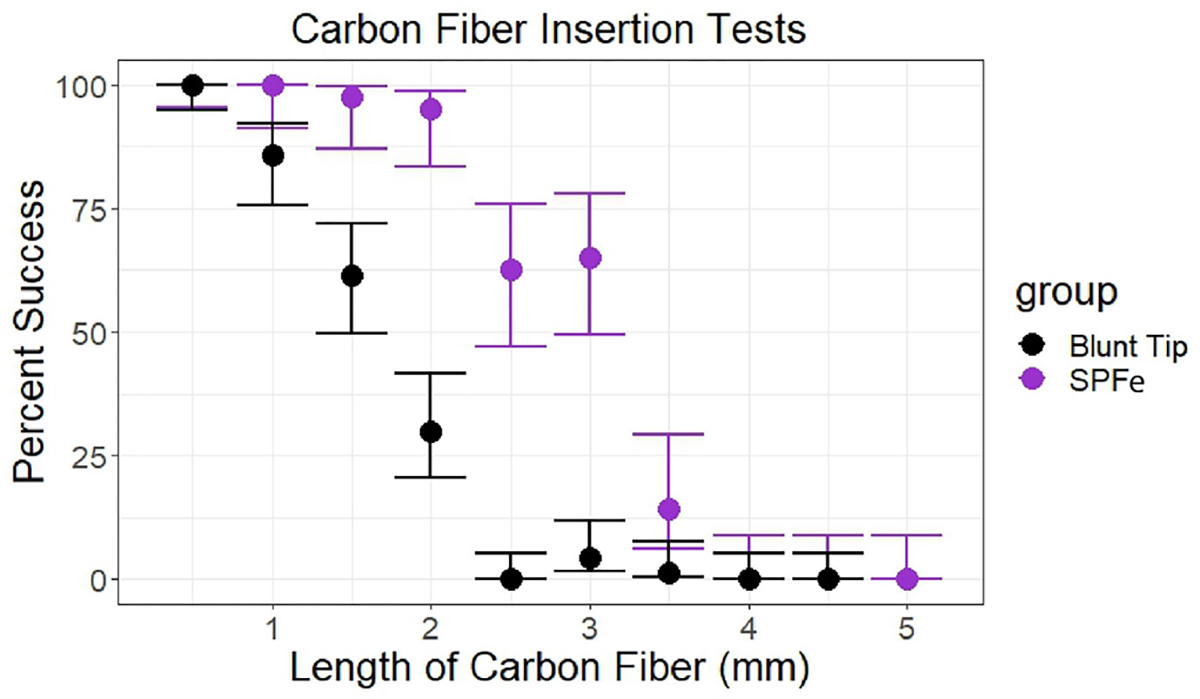
Successful insertion of the carbon fiber into rat brain. Sharpened fibers not only penetrate deeper into the cortex than blunt fibers, but also change the insertion profile of the carbon fiber as a chi square test shows a p <2e-6 significant difference for the confidence intervals at each point in the comparison. For lengths 0.5–5 mm blunt n = 80, for SPFe n = 40, except at lengths 3.5 mm and 0.5 mm, which were n = 35 and n = 80 respectively.

To evaluate SPFe insertion capabilities, SPFe fibers were inserted into rodent brain acutely following techniques from previous literature [50]. We compared the insertion profile of SPFe to historical data collected from blunt tipped carbon fibers [50]. Blunt carbon fiber electrodes could insert into brain without aid of a shuttle or surgical intervention with a 100% success rate at a length of 500 *μ*m. The SPFe tips extend that ‘self-insertion’ length to 1.5 mm (p < 2e-6). Overall, the SPFe tips were usually able to insert into the brain at < 2 mm lengths (Figure 6). A Wilson Binomial Confidence Interval Test was used to compare blunt (n = 70) and SPFe tips (n at least 35). A Pearson’s Chi-Squared Test was used to determine significant differences between the groups. A p-value of p < 2e-6 indicated that sharpening the fiber changes the insertion profile at all lengths. Sharpening the tips therefore allows fibers to insert more easily to cortical depths deeper than Layer 3 without need of a shuttle.

**Fig. 7.**
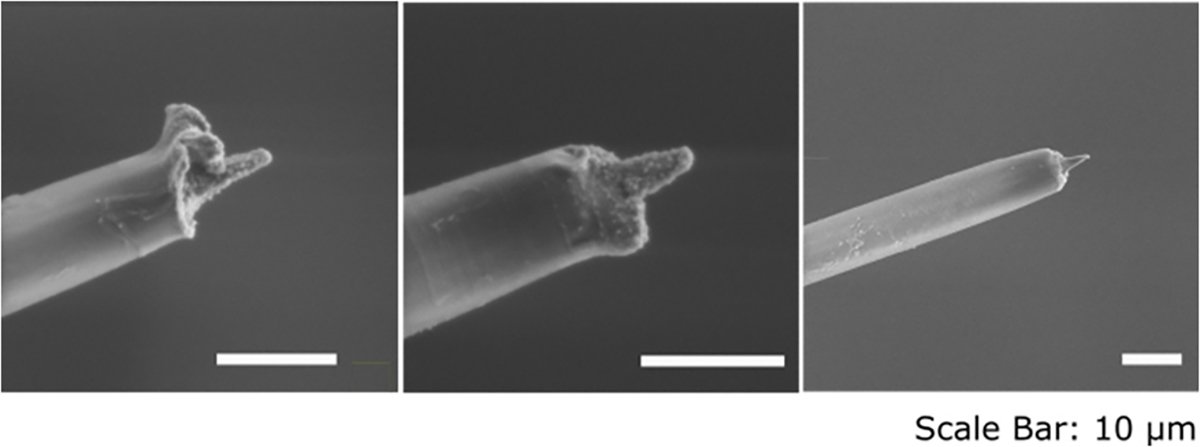
Example SEM images of explanted SPFe after being inserted to depths of 1.5mm in rat cortex. Note the geometry of the tip is still present and the PEDOT:pTS can be observed (rough texture on the tips).

As the SPFe tips are much thinner than the rest of the fiber (1–2 *μ*m at the tip vs 8 *μ*m along the insulated fiber), inserted electrodes (n = 8) were inspected to ensure that the tips had been robust enough to survive surgical insertion. The arrays were explanted, cleaned, and imaged using SEM. Figure 7 shows three representative functionalized fibers explanted from rat cortex. The tips maintained not only their pointed geometry but the PEDOT:pTS coating as well. This suggests the SPFe tips can survive implantation and maintain the small, sharpened tip that is desirable for recording electrodes.

### D. In-Vivo Viability

**Fig. 8.**
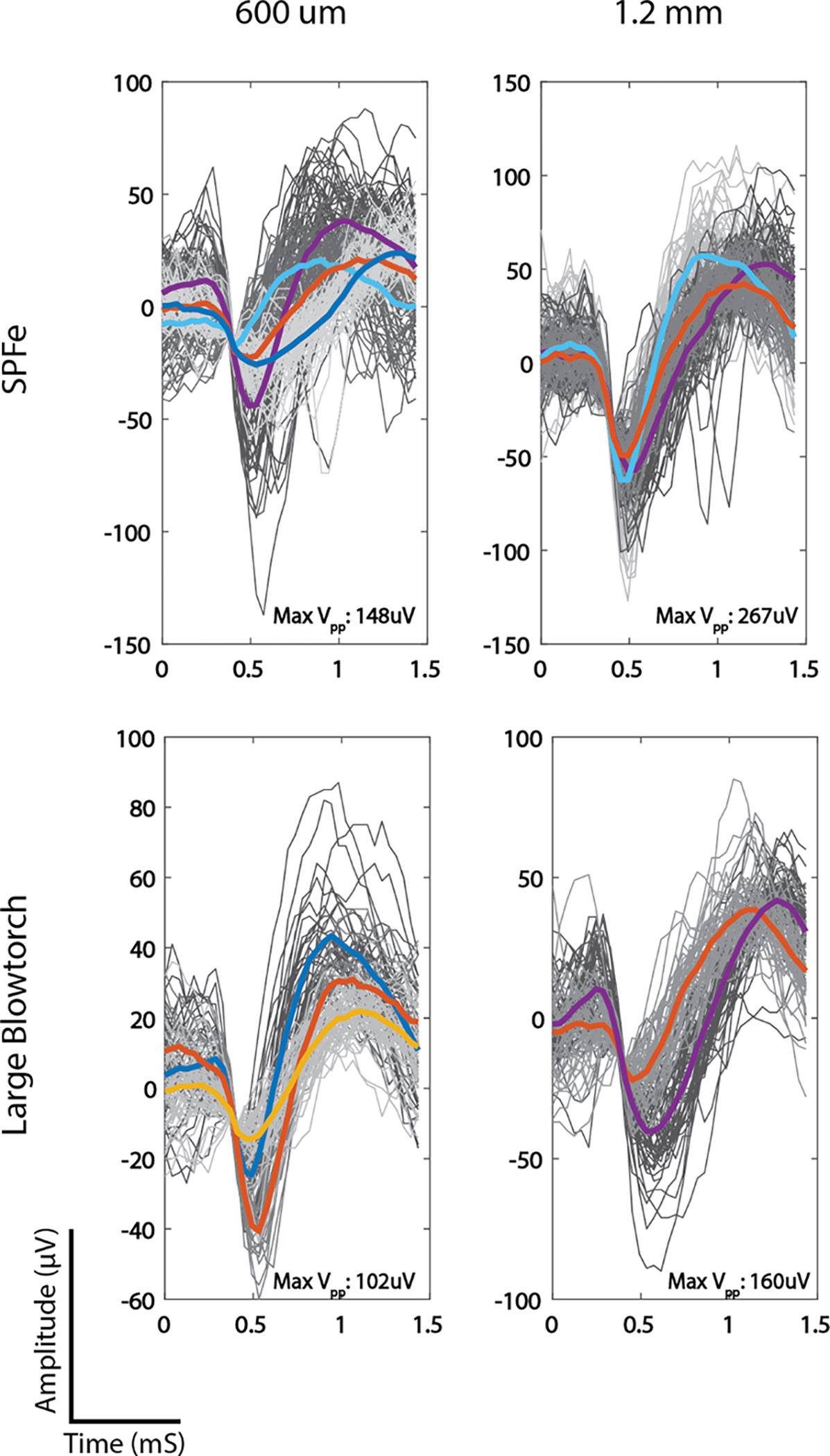
Rat motor cortex spikes from simultaneously inserted large blowtorch and SPFe tips at 600 *μ*m and 1.2 mm. The maximum peak-to-peak voltage for the representative electrode shown is listed in the bottom right corner of the plots. The SPFe has significantly larger units at both locations.

Recordings were obtained from an array with both large-blowtorch and SPFe tips (n = 8 each) for direct comparison in performance. The array was inserted into rat motor cortex from 400 – 1600 *μ*m in 200 *μ*m steps. Yield for both electrode type is reported with the average amplitude across the electrodes that were able to record at a given depth. Yield is reported as electrodes with spikes larger than 50 *μ*V/number of working electrodes. Representative waveforms from 600 *μ*m and 1.2 mm depths can be seen in Figure 8 as proof-of-concept from 2 arrays. Overall, SPFe tips had higher yield and higher average peak-to-peak waveform values at both 600 *μ*m (158.13 ± 38.03 *μ*V, yield = 8/8) and 1.2 mm (249.5 ± 123.90 *μ*V, yield = 15/16) than the large-blowtorch probes at the same depths (99.4 ± 25.24 *μ*V, yield= 5/8; 158.13 ± 38.03 *μ*V yield = 2/16). The p-value for a one-tailed T-test at 600 *μ*m was p < 0.005, and for 1.2 mm p < 0.05. This indicates a significant increase in amplitudes recorded from the SPFe compared to the large-blowtorch fiber, which is expected due to averaging across less of the electric field from each neuron.

**Fig. 9.**
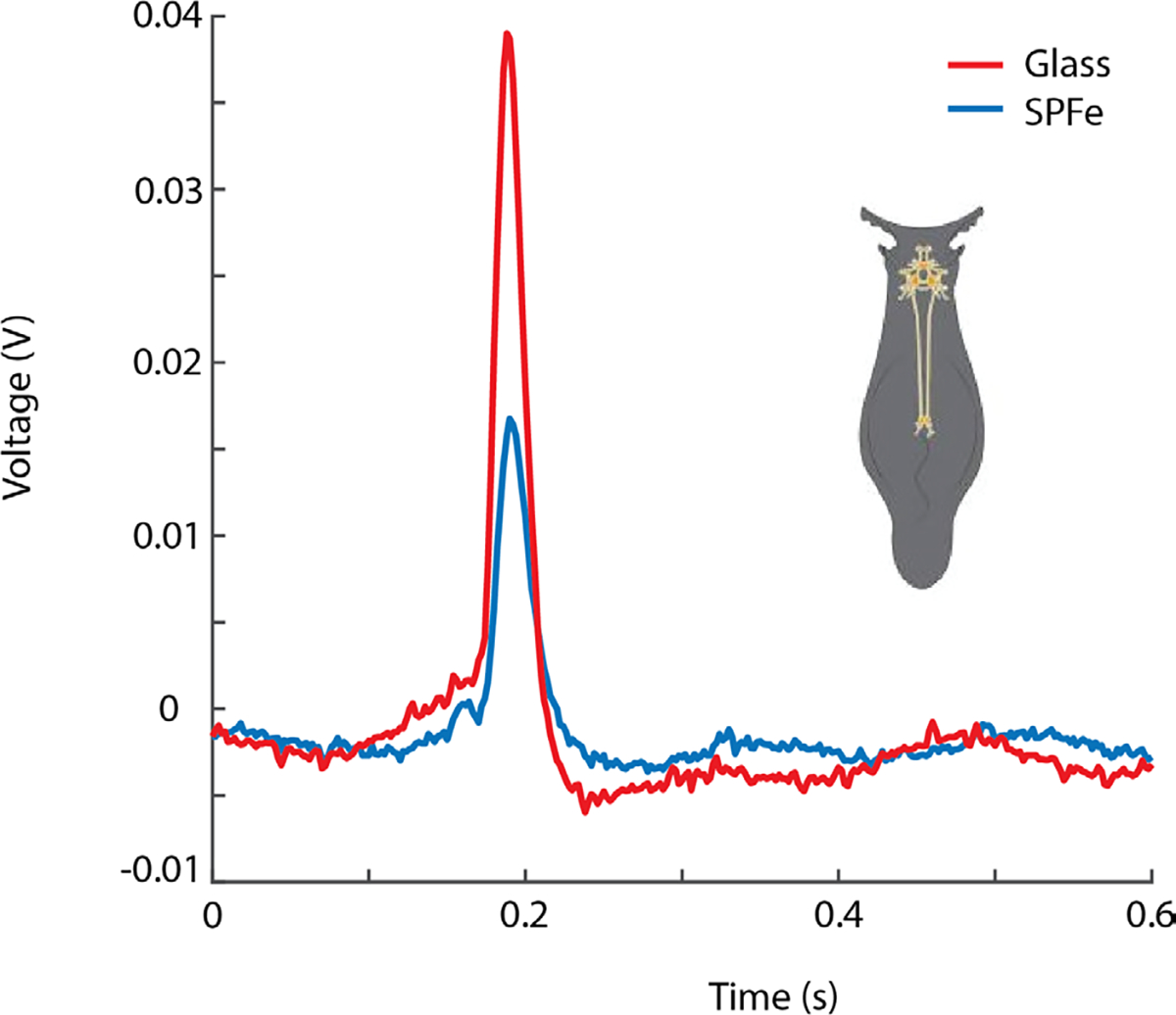
Spikes recorded after stimulating an Aplysia neurons. The spike amplitude of the SPFe (blue) was smaller than but comparable to that of the glass microelectrode (red).

*Aplysia* neurons are relatively large and easier to access, so they were used for testing the intracellular recording ability of SPFe. Intracellular action potentials on the SPFe were reliably recorded from several *Aplysia* motor neurons and ranged in amplitude from 15 – 27 mV. In one case, the SPFe was also compared to a traditional glass microelectrode to test its intracellular recording ability. Figure 9 shows a representative spike from a glass electrode and SPFe inside of the same neuron. The average amplitude recorded by the SPFe was 18.2 ± 1.4 mV (n = 36). The average amplitude recorded by the glass microelectrode was 41.4 ± 2.7 mV (n = 36). Although the goal was a direct comparison, multiple penetrations of the cell membrane could damage the integrity of the cell and decrease the recorded membrane potential. When stimulating as the only electrode in the neuron, the SPFe action potentials were observed at 25.4 ± 1.0 mV (n = 15). The addition of a glass microelectrode into the same neuron reduced the stabilized SPFe amplitude to 18.2 ± 0.8 mV (n = 15). The same action potentials recorded by the glass microelectrode stabilized at 40.2 ± 0.8 mV (n = 15). Overall, the action potential amplitude recorded by SPFe was smaller than that recorded by a traditional glass microelectrode, but was still sufficient to clearly discriminate intracellular spikes.

Peripheral recordings are another difficult recording challenge since the tissue that binds and protects nerve fibers is tough to penetrate [45], [56]. SPFe arrays coated in PEDOT:pTS were implanted into octopus ANC to test SPFe penetration and recording in this model. The octopus arm was removed from the body and the ANC was exposed before inserting an array of SPFe. The arm was stimulated physically and the recorded response was analyzed using Spike2 and MATLAB. Spike panels from the analysis (Figure 3) show several representative spikes from each stimulation recording paradigm. The largest recorded spike had a peak-to-peak amplitude of 111.4 *μ*V. Previous attempts recording from octopus using the large-blowtorch method resulted in noisy signals and no detectable waveforms. By reducing the size of the electrode, units could be identified. This indicates that the SPFe can be used *in vivo* and record reliably in nerve-like structures.

### E. Intraretinal Stimulation

**Fig. 10.**
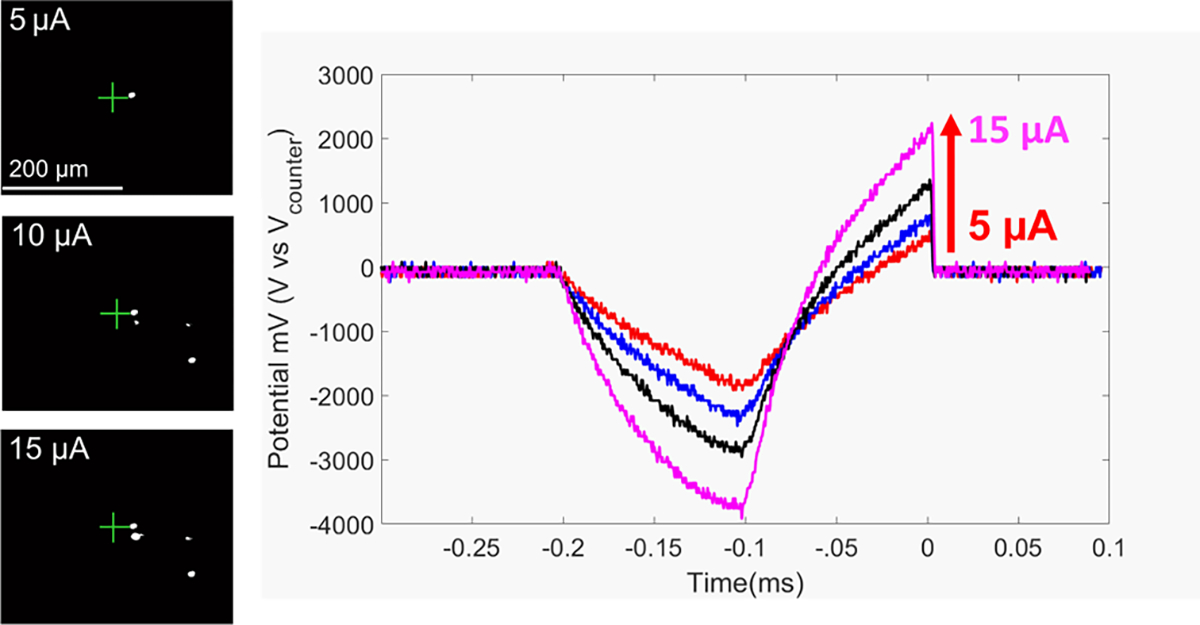
Resolution of the stimulation from SPFe can be seen (left). The green cross hair denotes the tip of the carbon fiber. The white indicates RGC activation in response to stimulation. Single cell resolution can be achieved at low stimulation amplitudes. Recorded voltage transients are shown on the right side when applying a current of 5, 7, 10, and 15 *μ*A.

Retinal stimulation was explored with SPFe. Wild-type mice retina were extracted and perfused on a transparent chamber. Carbon fibers were inserted such that the tips were 20 *μ*m from the RGC from the photoreceptor side. A train of pulses were applied to the retina and RGCs, and the resulting fluorescence images were recorded with an EMCCD camera. Figure 10 (left panel) shows change in fluorescence evoked by stimulation pulses of 5, 10, and 15 *μ*A amplitude. Single RGC resolution was achieved with the SPFe, indicating an extremely selective stimulation electrode. Voltage transients (VT) were recorded (Figure 10, right panel), for PtIr coated SPFe, when stimulating the retina at different current levels of 5, 7, 10, and 15 *μ*A. The shape of the VT is typical for stimulation electrodes [57] and the lack of a distorted waveform is an indicator that no significant hydrolysis occurred when applying these pulses to the SPFe.

The VT traces reported in Figure 10 represent how increasing the current amplitude increases the voltage across the electrode interface. VTs were monitored during the stimulation process to ensure no loss of coating occurred. Finally, EIS before and after each stimulation confirmed electrode stability (data not shown).

## IV. DISCUSSION

We demonstrate fabrication and validation of the novel SPFe version of the carbon fiber electrode. Our prior work included carbon fibers with small electrode area but blunt tips and separately carbon fibers with sharpened tips, but larger electrode area. The former enabled precise unit recording while the latter improved penetration into neural tissue. SPFe incorporates those two desirable features into a single device. These SPFe showed the capability to record *in vivo* from small structures (Figures 4, 8, 9). This expands the experimental applications of carbon fibers to smaller neural structures that have been difficult to record from previously. One such example is insect nervous systems. Honeybee [70], [71] and dragonfly [72] researchers trying to decode different insect behaviors struggle with finding electrodes small enough to insert without damaging the fragile insect body. Carbon fibers would be an excellent option for these systems, and adding the SPFe tips would allow for selective recording in these already very tiny systems.

While the SPFe opens the door to new non-mammalian models, it can improve upon existing neural interfaces used in mammalian models as well. Targeting specific cells with retinal prostheses has been a challenge. Distance between the electrodes and target cells, large electrode size, and unintended stimulation of axons can lead to off-target RGC activation and a lower image resolution [62], [73], [74], [75]. Multiple studies have investigated intraretinal electrodes to increase stimulation precision with retinal implants. One study placed a glass pipette in the retina to evoke RGC responses [76]. While this gave information on the magnitude of stimulation needed for intraretinal electrodes, glass pipettes are impractical for prostheses and placement was not well controlled. Another study showed biocompatibility and functional testing of 10*μ*m pillar electrodes with 55 *μ*m and 40 *μ*m pitch on a subretinal implant. Migration of retinal tissue into the space between pillars was noted [77] and stimulus threshold was decreased [78]. Pillar height of 10*μ*m limited the amount of penetration into the retina, but reduced proximity to the target cells and threshold by 78%. Our prior work using blowtorched carbon fibers also show decreased threshold [63]. The NR600 is an experimental retinal prosthesis with an array of penetrating electrodes. This intraretinal prosthesis was implanted in 9 patients [79]. The intraretinal array includes 25 *μ*m diameter fibers spaced at 100 *μ*m. The perceptual thresholds averaged 1.3 nC, which is significantly lower than epiretinal implants. Carbon fibers allow for stimulation [57] and their strength at small diameter allows penetration into the retina. With respect to the NR600 form factor, using carbon fibers would reduce the cross section of the penetrating electrode shanks to 13% of the existing size. The SPFe’s small surface area and charge storage capacity shown in this paper may allow for selective stimulation down to single cell resolution while the small diameter of the carbon fibers will minimize damage to the retina. The small electrode surface area creates challenges for electrochemical measurements. Cyclic voltammagrams were acquired at higher scan rates than is typical, to increase the signal (vs. noise, see Figure 5 for noise on bare carbon fiber). Voltage transients did not show clear demarcations to allow E_mc_ measurements [5]. Thus, comparisons of SPF stimulation performance via accepted figures of merit will require further experimentation. Nevertheless, we demonstrated electrically elicited retinal responses using SPF coated with PtIr. This opens the possibility for high-density retinal implants that are necessary to achieve improved visual acuity. While carbon fiber arrays are difficult to build, especially in high density configurations, many groups are working on automated placement [80], [81] to make these arrays more viable as commercial products.

Similarly, Layers 1–3 of cortex present some of the same challenges as retina. While much of epilepsy work relies on surface stimulation and recording, having a penetrating electrode might allow for better resolution in pre-ictal detection and response [82], [83]. However, cortical layers 1–3 contain small neurons from which it is difficult to record. SPFe could provide the ability to penetrate into brain and record from neurons with small cell bodies. While the arrays in this paper were linearly placed, alternative backend connectorization for carbon fibers [40], [84] has been explored previously and combining these approaches would be straightforward.

More difficulties lie in recording from tougher tissues like nerve that are also usually embedded in actively moving muscles. Previous work from our lab [45], [51], [85] has shown that carbon fibers can become more robust when adding a thin layer of silicone rubber to the base of the carbon fibers as it reduces the shear forces at the interface of the fiber and the board. Welle et al., show that these fibers are capable of recording from feline DRG [45]. As was seen in the octopus data, having a smaller surface area probe allowed for units to be recorded. As octopus axon cords have no myelination [86], recordings from mammalian nerves with myelination may also be improved due to the small surface area of the SPFe.

While this study has a plethora of preliminary data in a number of animal models, there is still optimization to be done. The PEDOT:pTS deposition technique follows previous methodology [56] that was optimized for a larger site electrode. While we did not see significant geometrical changes of the tip under SEM, this process could be further optimized for smaller site deposition. While we found PEDOT:pTS to be unstable in stimulation and switched to PtIr, other groups have found success with PEDOT:pTS for stimulation [87], [88]. The lack of stability for PEDOT:pTS may lie in the deposition method presented here and will be further examined in future work.
